# Molecular Characterization of Antimicrobial Resistance in* Escherichia coli* from Rabbit Farms in Tai'an, China

**DOI:** 10.1155/2018/8607647

**Published:** 2018-02-26

**Authors:** Xiaonan Zhao, Jie Yang, Zijing Ju, Weishan Chang, Shuhong Sun

**Affiliations:** College of Animal Science and Technology, Shandong Agricultural University, Tai'an 271000, China

## Abstract

To investigate the prevalence and resistance against antimicrobials of* Escherichia coli (E. coli)* in Tai'an, March 2016, a total of 55* E. coli* strains were isolated from 60 faecal samples of diarrheic rabbits collected from three rabbit farms in Tai'an. The* E. coli* isolates were assayed for antimicrobial susceptibility and prevalence of resistance genes and Class I integrons and genotyped using Multilocus Sequence Typing (MLST). All the* E. coli* isolates were sensitive to ceftazidime, ceftriaxone, imipenem, and amikacin, while 78.2% of the isolates showed resistance against tetracycline, and 65.5% were resistant against ampicillin. The most common resistance gene detected was *bla*_TEM_, present in 98.2% of isolates, followed by *bla*_CTX-M_ (94.6%) and* sul2* (58.2%). Class I integrons were detected in 17 out of the 55 (30.9%)* E. coli* strains. Seven kinds of gene cassette were detected:* dfrA17* +* aadA5*,* dfrA1* +* catB3* +* aacA4*,* aadA2* +* LinF*,* dfrA1* +* aadA1*,* aadA22*,* dfrA12* +* orfF* +* addA2*, and* aadA16* +* dfrA27* +* arr-3*. All the 55* E. coli* strains were identified and classified as 13 sequence types (STs); ST302 (22/55, 40.0%) was the most prevalent type, followed by ST370 (12/55, 21.8%). This study showed that* E. coli* isolated from diarrheic farmed rabbits in the Tai'an area exhibit sometimes very frequent resistance to antimicrobials important to human medicine, which further highlights the need for reasonable use of antibiotics.

## 1. Introduction

Antimicrobials, which play a crucial part in animal and human health care, are commonly used to treat bacterial infections and to prevent further spread of the infections within animal groups [1, 2].* E. coli* are commensal bacteria colonizing the intestinal tract of rabbits and other warm-blooded animal species [3]. They may frequently be exposed to selection pressure caused by antimicrobial treatments and may contribute considerably to the emergence of antimicrobial resistant bacteria and the spread of antimicrobial resistance [4]. Furthermore, antimicrobial resistant bacteria can be transferred to the humans through the food chain, thus affecting human health.

There are many kinds of molecular mechanisms underlying antimicrobial resistance in bacteria. The mechanisms mediated by integrons are an example of the most studied ones. Integrons, one kind of genetic element, are able to capture exogenous genes and disseminate them through plasmids or transposons in both interspecific and intraspecific ways [5].

Genotyping methods of bacteria have been important in surveillance and analysis, and they enable determination of sources of outbreaks. MLST is based on DNA sequencing data, which makes it specific, repeatable, comparable, and suitable for detecting the evolution of the strains and genetic analysis of microbial populations [6, 7].

Most previous studies were focused on the prevalence of antimicrobial resistance of* E. coli* in the main food animal species such as chicken and pigs, but there is a lack of research on antimicrobial resistance in rabbits. Therefore, in this study, we collected faecal samples from rabbit farms in the Tai'an area and isolated* E. coli* strains and then analyzed their drug resistance characteristics and the genotypes, in order to provide information to determine reasonable use of antibiotics.

## 2. Materials and Methods

### 2.1. Description of Sampling Sites

From February to May 2016, 60 faecal samples (20 samples per farm) were randomly collected from diarrhea rabbits on farms in Ningyang, Xintai, and Dongping regions in Tai'an, China. The samples were independently collected from individual animals and the farms were chosen based on their scale (the breeding stock was >1500 heads). According to current statistics, there are about 15 relatively large rabbit farms in Tai'an, and each farm presumably produces >4000 heads annually. Because the sampling process did not harm the animals, ethical approval was not required for the study.

### 2.2. Samples and* E*.* coli* Isolation

Isolation and identification of* E. coli* were performed as described previously [8], with some modifications. Briefly, about 0.5 g faecal samples were added to a tube containing 5 mL of LB (Luria-Bertani) medium and cultured at 37°C for 12 h. The bacteria solution was then streaked onto eosin-methylene blue agar (EMB; Hope, Qingdao, China) plates. After incubation at 37°C for 18~24 h, typical colonies on the plate were streaked on MacConkey's agar (MAC; Hope, Qingdao, China) plates. Positive colonies were chosen for further biochemical identification using the API 20E system (Sysmex bioMérieux, Tokyo, Japan).

### 2.3. Antimicrobial Susceptibility Testing

Antimicrobial susceptibility was tested by the Minimal Inhibition Concentration (MIC) method. Bacteria were cultured at 37°C in the LB broth medium for 6 h. The concentration of* E. coli* was adjusted to 1.5 × 10^8^ colony forming unit (CFU)/mL in sterile saline. The susceptibility of the* E. coli* isolates was tested to 14 commonly used antimicrobial agents, including ciprofloxacin (CIP), chloramphenicol (C), nalidixic acid (NA), amoxicillin/clavulanic acid (AML), tobramycin (TB), ceftazidime (CAZ), ceftriaxone (CRO), gentamicin (GEN), sulfamethoxazole/trimethoprim (SXT), imipenem (IMP), tetracycline (TET), ampicillin (AMP), cefoxitin (FOX), and amikacin (AMK). The results were interpreted based on the Clinical and Laboratory Standards Institute (CLSI) standard guidelines [9]. In this study, the* E. coli* strain ATCC25922 was used as the quality control strain.* E. coli* isolates that were resistant to more than three classes of antimicrobials were defined as multidrug-resistant (MDR) isolates.

### 2.4. Detection of Resistance Genes

The* E. coli* DNA was extracted using Bacterial Genomic DNA Extraction Kit (Tiangen, Beijing, China) and then the resistance genes associated with beta-lactams (*bla*_TEM_, *bla*_SHV_, *bla*_OXA_, *bla*_PSE_, and *bla*_CTX-M_), the quinolone-resistance genes* (qnrA*,* qnrB*,* qnrS*,* aac(6)-Ib-cr*, and* qepA)*, the aminoglycosides-resistance genes (*aac(3)-I*,* aac(3)-II*,* aac(3)-III*,* aac(3)-IV*,* ant(2)*, and* aac(6)-Ib*), the tetracycline-resistance genes* (tetA* and* tetB)*, the sulfonamides-resistance genes* (sul1*,* sul2*, and* sul3)*, and the chloramphenicol-resistance genes* (cmlA* and* flor)* were amplified by PCR, using the previously reported [10–12] primers and conditions, and the gene sequences were listed in Table 1. In addition, the PCR procedure was repeated twice for all the isolates, and PCR products were analyzed by 1.5% agarose gel electrophoresis at 100 V for 1 h. Photos were taken with a gel imaging system (Tanon-2500, Shanghai, China).

### 2.5. Detection of Integrons

The universal primers for the amplification of Class I integron gene cassette genes were detected by PCR. The primers of cassette FP and cassette RP were designed according to reference: 5′-TCATGGCTTGTTATGACTGT-3′ and 5′-GTAGGGCTTATTATGCACGC-3′. The amplification consisted of an initial denaturation at 94°C for 5 min, 30 cycles of denaturation at 94°C for 60 s, annealing at 56°C for 55 s, and extension at 68°C for 6 min. A final extension for 10 min at 72°C was also applied. PCR products were purified and sequenced for the further analysis [13].

### 2.6. MLST

According to http://bigsdb.pasteur.fr/ecoli/primers_used.html, 8 pairs of primers for housekeeping genes (dinB, icdA, pabB, polB, putP, trpA, trpB, and uidA) were designed and then used for PCR. The products of PCR amplification were sequenced by Shanghai Sangon Biotech Co., Ltd. and the results were amended using the Chromas and DNA Star software and then submitted to the Pasteur online database (http://bigsdb.pasteur.fr/perl/bigsdb/bigsdb.pl?db=pubmlst_ecoli_seqdef_public&page=sequenceQuery) for processing. Then the allele number of each housekeeping gene was obtained and the sequence type (ST type) of each strain was acquired.

## 3. Results

### 3.1. Isolation of* E. coli*

A total of 55* E. coli* strains were isolated from 60 samples: 18 strains were from Ningyang, 20 strains were from Xintai, and 17 strains were from Dongping (Table 3).

### 3.2. Antimicrobial Susceptibility Test

From Table 2, all the strains were sensitive to ceftazidime, ceftriaxone, imipenem, and amikacin. The highest resistance rate was shown against tetracycline (46/55, 78.2%) and ampicillin (36/55, 65.5%). The two most common antimicrobial resistance profiles of the isolates were AMP-NA-TET (*n* = 10, 18.1%) and AMP-TET (*n* = 8, 14.5%). In addition, 6 strains were sensitive to all the 14 kinds of antibiotics, while there were 50.9% (28/55) multidrug-resistant strains (Table 3).

### 3.3. Detection of Resistance Genes

Two kinds of *β*-lactamase genes, *bla*_TEM_ and *bla*_CTX-M_, were the most frequently detected genes at 98.2% and 94.5% respectively, followed by* sul2*, a kind of sulfa drug-resistant gene, with the detection rate of 58.18%. 5 (9.1%) strains had* tetB*, a tetracycline-resistance gene. 3 strains (5.5%) had shown resistance to* qnrS* and 4 strains (7.5%) to* aac(6)-Ib-cr*, which were quinolone-resistance genes (Table 3).

### 3.4. Detection of Integrons

Class I integrons were detected in 17* E. coli* isolates out of 55 strains, as the detection rate was 30.9%. Seven kinds of drug-resistance gene cassette were found in those Class I integrons as follows:* dfrA17* +* aadA5*,* dfrA1* +* catB3* +* aacA4*,* aadA2* +* LinF*,* dfrA1* +* aadA1*,* aadA22*,* dfrA12* +* orfF* +* addA2*, and* aadA16* +* dfrA27* +* arr-3*. The most prevalent gene cassettes were* dfrA1* +* catB3* +* aacA4* and* aadA2* +* LinF* (Table 3).

### 3.5. MLST

13 different kinds of STs were identified among all the 55 strains, including 3 STs (ST302, ST468, and ST370) from samples derived from Ningyang, 6 STs (ST87, ST302, ST314, ST370, ST468, and ST636) from samples derived from Xintai, and 8 STs (ST2, ST24, ST88, ST353, ST370, ST461, ST731, and ST739) from samples derived from Dongping. It was shown that ST302 (22/55, 40.0%) was the most prevalent type, followed by ST370 (12/55, 21.8%) (Table 3).

## 4. Discussion

Colibacillosis of rabbits, a common disease in rabbits breeding, had become one of the major infectious diseases that endanger the rabbit breeding industry [14, 15]. In this study, the highest resistance rates of 55* E. coli* strains were against tetracycline and ampicillin (78.2% and 65.5%, resp.), higher than those reported from wild rabbits in Portugal, in which the tetracycline and ampicillin resistance rates were both 11.4% [16]. This coincided with the fact that tetracycline and ampicillin have been widely used to control and prevent rabbit disease in the Tai'an area. In this study, all the strains were sensitive to ceftazidime, ceftriaxone, imipenem, and amikacin; this may be due to the less frequent use of these antibiotics in rabbit farms. The occurrence of MDR strains is a potential threat to the public health. Our data showed that the proportion of MDR strains among all the* E. coli* strains was 50.9%, which was lower than that observed from wild rabbits (71.4%) [16], which may be due to the fact that wild rabbits and hares are commonly found in areas inhabited by human beings and livestock and consequently may share common sources of exposure to antibiotics.

*bla*_TEM_, one kind of *β*-lactamase genes, is the most common mechanism of ampicillin resistance in* E. coli*, and it was previously shown in ampicillin-resistant* E. coli* isolates from foods, humans, and healthy animals in Spain [17]. In this study, 98.2% of* E. coli* isolates carried *bla*_TEM_, whereas only 61.8% were resistant to ampicillin; this may be associated with the expression status of *bla*_TEM_ genes and is needed to be further studied. Extended-spectrum cephalosporins are critically important antibiotics in human medicine [18], which are also used to treat food-producing animals. The acquirement of extended-spectrum cephalosporins resistance was mainly mediated by extended-spectrum *β*-lactamases in* E. coli*, while the predominant ones were the CTX-M [19]. In this study, 94.5% of* E. coli* isolates carried *bla*_CTX-M_, whereas no strains were resistant to cephalosporins; the reason may be that the resistance genes had not yet led to the resistance phenotype. Phenotype could be controlled and verified with the MIC methods. In addition, the quinolone-resistance genes,* qnrS* (5.5%) and* aac(6)-Ib-cr* (7.5%), were much less frequent than the report in Sichuan, where* aac(6)-Ib-cr* (80.4%) and* qnrS* (59.8%) were observed [20]; the difference may be the result of the less frequent use of fluoroquinolone in the Tai'an area, while the detection of the quinolone-resistance genes in this study may come from other sources, such as the contaminated water and food. It has been reported that* tetA* and* tetB* are associated with tetracycline resistance from human and animal sources [21], but our results differed in that tetracycline-resistant* E. coli* in our rabbit were not all carrying* tetA* or* tetB*; this may be related to regional differences and different breeding conditions. With regard to chloramphenicol-resistant strains, most of them were carrying* flor* genes; however,* cmlA* genes were not very common among chloramphenicol-resistant strains, which was different from other reports, where the* flor* and* cmlA* genes occurred with the same percentage [10]; this difference may be the result of the fact that only partial genes were expressed. The* sul1* and/or* sul2* and/or* sul3* genes were detected in all of the sulfamethoxazole/trimethoprim-resistant* E. coli* isolates obtained from rabbits.

In this study, Class I integrons were detected in 17* E. coli* isolates out of 55 strains (30.91%), which was similar to the percentage (31.5%) of a previous study conducted by Hai et al. [22] but is lower than that of a previous study observed by Dotto et al. (2014) who detected 61.1% of* E. coli* strains isolated from domestic and wild Lagomorphs in northern Italy between 2006 and 2008 carrying Class I integrons [23]. Of note, Class I integrons are often associated with MDR* E. coli* isolates, consistent with the result of the present study.

In this study, the highest isolation rate of 40% (22/55) was found for ST302 which had not yet been reported related to infections. It was different from that of previous studies which found that ST40 is the most common ST from rabbits in Italy [23].

## 5. Conclusions

Our findings exhibit the molecular characterization of antimicrobial resistance in* Escherichia coli* from rabbit farms in Tai'an, China. The rabbits in farms are contaminated with MDR* E. coli*, which may originate from farms further up the food chain or from horizontal contamination. The high detection rates of MDR* E. coli*, Class I integrons, and antibiotic-resistance genes suggested that measures should be taken to reduce the harm to public health, such as the reasonable use of antimicrobials in animal husbandry and culling the reservoirs of pathogens from the slaughter process.

## Figures and Tables

**Table 1:** Primers for PCR in this study.

Primer name	Sequence (5′→ 3′)	Reference
*bla*_TEM_	F: ATTCTTGAAGACGAAAGGGC	[10]
	R: ACGCTCAGTGGAACGAAAAC	
*bla*_SHV_	F: CACTCAAGGATGTATTGTG	[10]
	R: TTAGCGTTGCCAGTGCTCG	
*bla*_OXA_	F: ACACAATACATATCAACTTCGC	[10]
	R: AGTGTGTTTAGAATGGTGATC	
*bla*_PSE_	F: TTT GGT TCCGCG CTA TCT G	[10]
	R: TAC TCC GAG CAC CAA ATC CG	
*bla*_CTX-M_	F: CGCTTTGCGATGTGCAG	[11]
	R: ACCGCGATATCGTTGGT	
*qnrA*	F: ATTTCTCACGCCAGGATTTG	[12]
	R: TGCCAGGCACAGATCTTGAC	
*qnrB*	F: CGACCTKAGCGGCACTGAAT	[12]
	R: GAGCAACGAYGCCTGGTAGYTG	
*qnrS*	F: ATGGAAACCTACAATCATAC	[12]
	R: AAAAACACCTCGACTTAAGT	
*acc(6′)-Ib-cr*	F: TTGCGATGCTCTATGAGTGGCTA	[12]
	R: CTCGAATGCCTGGCGTGTTT	
*qepA*	F: GCAGGTCCAGCAGCGGGTAG	[12]
	R: CTTCCTGCCCGAGTATCGTG	
*aac(3)-I*	F: ACCTACTCCCAACATCAGCC	[10]
	R: ATATAGATCTCACTACGCGC	
*aac(3)-II*	F: ACTGTGATGGGATACGCGTC	[10]
	R: CTCCGTCAGCGTTTCAGCTA	
*aac(3)-III*	F: CACAAGAACGTGGTCCGCTA	[10]
	R: AACAGGTAAGCATCCGCATC	
*aac(3)-IV*	F: CTTCAGGATGGCAAGTTGGT	[10]
	R: TCATCTCGTTCTCCGCTCAT	
*ant(*2^″^)	F: ATGTTACGCAGCAGGGCAGTCG	[10]
	R: CGTCAGATCAATATCATCGTGC	
*acc(6′)-Ib*	F: TTGCGATGCTCTATGAGTGGCTA	[10]
	R: CTCGAATGCCTGGCGTGTTT	
*tetA*	F: GCGCCTTTCCTTTGGGTTCT	[10]
	R: CCACCCGTTCCACGTTGTTA	
*tetB*	F: CATTAATAGGCGCATCGCTG	[10]
	R: TGAAGGTCATCGATAGCAGG	
*sul1*	F: TGGTGACGGTGTTCGGCATTC	[10]
	R: GCGAGGGTTTCCGAGAAGGTG	
*sul2*	F: CGGCATCGTCAACATAACC	[10]
	R: GTGTGCGGATGAAGTCAG	
*sul3*	F: CATTCTAGAAAACAGTCGTAGTTCG	[10]
	R: CATCTGCAGCTAACCTAGGGCTTTGGA	
*cmlA*	F: TGTCATTTACGGCATACTCG	[10]
	R: ATCAGGCATCCCATTCCCAT	
*flor*	F: CTGAGGGTGTCGTCATCTAC	[10]
	R: GCTCCGACAATGCTGACTAT	

**Table 2:** Susceptibility of *E. coli* to 14 commonly used antibiotics (S: susceptible; I: intermediate; R: resistant).

Antibiotics	Disk diffusion [*n* (%)]		
	S	I	R
AML	53 (96.4%)	0	2 (3.6%)
AMK	55 (100%)	0	0
AMP	15 (27.3%)	4 (7.3%)	36 (65.5%)
C	41 (74.5%)	3 (5.5%)	11 (20.0%)
CAZ	0	0	0
CIP	43 (78.2%)	2 (3.6%)	10 (18.2%)
CRO	0	0	0
FOX	54 (98.2%)	0	1 (1.8%)
GEN	46 (83.6%)	3 (5.5%)	6 (10.9%)
IMP	0	0	0
NA	10 (18.2%)	15 (27.3%)	30 (54.5%)
SXT	35 (63.6%)	2 (3.6%)	18 (32.7%)
TB	46 (83.6%)	8 (14.5%)	1 (1.8%)
TET	8 (14.5%)	4 (7.3%)	43 (78.2%)

**Table 3:** Resistance phenotype, ST, incidence of Class I integrons, and resistance gens in *E. coli *isolated from rabbit farms.

Number	Location	ST	Resistance phenotype	Integrons/resistance
(1)	Ningyang	ST302	AMP-NA-TET	*bla*_CTX-M_, *bla*_TEM_, *sul2*
(2)	Ningyang	ST302	AMP-TET	*bla*_CTX-M_, *bla*_TEM_, *sul2*
(3)	Ningyang	ST302	AMP-NA-TET	Class 1 *(dfrA17* + *aadA5)*, *bla*_CTX-M_, *bla*TEM, *sul2*
(4)	Ningyang	ST302	AMP-TET	*bla*_CTX-M_, *bla*_TEM_, *sul2*
(5)	Ningyang	ST468	AMP-C-NA-SXT-TET	Class 1 *(dfrA17* + *aadA5)*, *bla*_CTX-M_, *bla*_TEM_, *flor*
(6)	Ningyang	ST302	AMP-CIP-NA-SXT-TET	*bla*_CTX-M_, *bla*_TEM_, *sul2*
(7)	Ningyang	ST468	AMP-C-NA-SXT-TET	Class 1 *(dfrA17* + *aadA5)*, *bla*_CTX-M_, *bla*_TEM_, *sul2*
(8)	Ningyang	ST302	AMP-TET	*bla*_CTX-M_, *bla*_TEM_, *sul2*
(9)	Ningyang	ST302	AMP-TET	*bla*_CTX-M_, *bla*_TEM_, *sul2*
(10)	Ningyang	ST468	AMP-CIP-NA-SXT-TET	*bla*_CTX-M_, *bla*_TEM_, *sul2*
(11)	Ningyang	ST302	AMP-TET	Class 1 (dfrA1 + aadA1), *bla*_CTX-M_, *bla*_TEM_, *sul2*
(12)	Ningyang	ST370		*bla*_CTX-M_, *bla*_TEM_
(13)	Ningyang	ST370		*bla*_CTX-M_, *bla*_TEM_, *sul1*
(14)	Ningyang	ST302	AMP-TET	*bla*_CTX-M_, *bla*_TEM_, *sul1*, *sul2*
(15)	Ningyang	ST370		*bla*_CTX-M_, *bla*_TEM_
(16)	Ningyang	ST302	AMP-NA-TET	*bla*_CTX-M_, *bla*_TEM_, *sul1*, *sul2*
(17)	Ningyang	ST302	AMP-TET	*bla*_CTX-M_, *bla*_TEM_, *sul2*
(18)	Ningyang	ST370		*bla*_CTX-M_, *bla*_TEM_
(19)	Xintai	ST370	SXT	*bla*_CTX-M_, *bla*_TEM_, *sul1*
(20)	Xintai	ST87	GEN-SXT	Class 1 *(dfrA17* + *aadA5)*, *bla*_CTX-M_, *bla*_TEM_, *qnrS*, *sul1*
(21)	Xintai	ST302	AMP-GEN-SXT-TET	*bla*_CTX-M_, *bla*_TEM_, *sul2*
(22)	Xintai	ST302	AMP-NA-TET	*bla*_CTX-M_, *bla*_TEM_, *sul2*
(23)	Xintai	ST370		*bla*_CTX-M_, *bla*_TEM_
(24)	Xintai	ST302		*bla*_CTX-M_, *bla*_TEM_, *sul1*
(25)	Xintai	ST314	NA-TET	Class 1 *(dfrA1* + *catB3* + *aacA4)*, *bla*_CTX-M_, *bla*_TEM_, *sul1*, *sul2*
(26)	Xintai	ST302	AMP-C-NA-SXT-TET	*bla*_CTX-M_, *bla*_TEM_, *flor*, *sul2*
(27)	Xintai	ST302	AMP-FOX-NA-TET	*bla*_CTX-M_, *bla*_TEM_, *sul2*
(28)	Xintai	ST302	TET	*bla*_CTX-M_, *bla*_TEM_, *sul2*
(29)	Xintai	ST302	AMP-NA-SXT	Class 1 *(aadA16* + *dfrA27* + *arr-3)*, *acc(6′)-Ib-cr*, *bla*_CTX-M_, *bla*_TEM_, *sul2*
(30)	Xintai	ST636		*bla*_CTX-M_, *bla*_TEM_, *sul1*
(31)	Xintai	ST370	AMP-NA-TET	*bla*_CTX-M_, *bla*_TEM_, *sul2*
(32)	Xintai	ST468	AMP-NA-TET	*bla*_CTX-M_, *bla*_TEM_,* sul2*
(33)	Xintai	ST468	AMP-C-NA-SXT	Class 1 *(dfrA1* + *catB3* + *aacA4)*, *bla*_CTX-M_, *bla*_TEM_, *flor*
(34)	Xintai	ST302	AMP-NA-TET	*bla*_CTX-M_, *bla*_TEM_, *sul2*
(35)	Xintai	ST302	AMP-NA-TET	*bla*_CTX-M_, *bla*_TEM_, *sul2*
(36)	Xintai	ST302	AMP-NA-TET	*bla*_CTX-M_, *bla*_TEM_, *sul2*
(37)	Xintai	ST302	AMP -TET	Class 1 *(dfrA1* + *catB3* + *aacA4)*, *bla*_CTX-M_, *bla*_TEM_, *sul2*
(38)	Xintai	ST370	NA-TET	Class 1 *(dfrA1* + *catB3* + *aacA4)*, *bla*_CTX-M_, *bla*_TEM_, *sul1*
(39)	Dongping	ST461	AMP-NA-SXT	*bla*_TEM_, *qnrS*, *sul1*
(40)	Dongping	ST731	TET	*bla*_TEM_
(41)	Dongping	ST739	AMP-NA-TET	*acc(6′)-Ib-cr*, *bla*_CTX-M_, *bla*_TEM_
(42)	Dongping	ST370	TET	*bla*_CTX-M_, *bla*_TEM_
(43)	Dongping	ST739	AMP-CIP- NA-SXT-TET	*acc(6′)-Ib-cr*, *bla*_CTX-M_, *bla*_TEM_
(44)	Dongping	ST370	TET	*bla*_CTX-M_, *bla*_TEM_
(45)	Dongping	ST88	AML-AMP-C-CIP-GEN- NA-SXT-TET	Class 1 *(aadA2* + *LinF)*, *bla*_CTX-M_, *bla*_TEM_, *cmlA*, *flor*, *sul2*, *sul3*,* tetB*
(46)	Dongping	ST370	TET	*bla*_CTX-M_, *bla*_TEM_, *sul1*
(47)	Dongping	ST739	AMP-CIP-NA-SXT-TET	*acc(6′)-Ib-cr*, *bla*_CTX-M_, *bla*_TEM_
(48)	Dongping	ST88	AMP-C-CIP-GEN-NA-SXT-TET	*bla*_CTX-M_, *bla*_TEM_, *cmlA*, *flor*, *sul2*, *sul3*, *tetB*
(49)	Dongping	ST2	AMP-C-CIP-NA-SXT-TET	*bla*_CTX-M_, *bla*_TEM_, *cmlA*, *flor*, *sul3*
(50)	Dongping	ST88	AMP-C-CIP-GEN-NA-SXT-TET	Class 1 *(aadA2* + *LinF)*, *bla*_CTX-M_, *bla*_TEM_, *cmlA*, *flor*, *sul2*, *sul3*, *tetB*
(51)	Dongping	ST353	C-TET	Class 1 *(aadA22)*, *bla*_TEM_, *flor*, *qnrS*, *sul2*
(52)	Dongping	ST370	NA-TET	Class 1 *(dfrA12* + *orfF* + *addA2)*, *bla*_CTX-M_, *sul1*
(53)	Dongping	ST88	C-CIP-NA-TET	Class 1 *(aadA2* + *LinF)*, *bla*_CTX-M_, *bla*_TEM_, *flor*, *sul2*, *sul3*, *tetB*
(54)	Dongping	ST24	AML-AMP-TET	Class 1 *(aadA22)*, *bla*_CTX-M_, *bla*_TEM_, *flor*, *sul1*
(55)	Dongping	ST88	AMP-C-CIP-GEN-NA-SXT-TB-TET	Class 1 *(aadA2* + *LinF)*, *bla*_CTX-M_, *bla*_TEM_, *cmlA*, *flor*, *sul2*, *sul3*, *tetB*
